# Glycyrrhizin Suppresses the Expressions of HMGB1 and Relieves the Severity of Traumatic Pancreatitis in Rats

**DOI:** 10.1371/journal.pone.0115982

**Published:** 2014-12-26

**Authors:** Ke Xiang, Long Cheng, Zhulin Luo, Jiandong Ren, Fuzhou Tian, Lijun Tang, Tao Chen, Ruiwu Dai

**Affiliations:** 1 The Third Military Medical University, Chongqing, P. R. China; 2 Department of General Surgery, Chengdu Military General Hospital, Chengdu, P. R. China; 3 Department of Pharmacy, Chengdu Military General Hospital, Chengdu, P. R. China; University of Szeged, Hungary

## Abstract

**Background:**

High mobility group box 1 (HMGB1) plays important roles in a large variety of diseases; glycyrrhizin (GL) is recognized as an HMGB1 inhibitor. However, few studies have focused on whether glycyrrhizin can potentially improve the outcome of traumatic pancreatitis (TP) by inhibiting HMGB1.

**Methods:**

A total of 60 male Wistar rats were randomly divided into three groups (n = 20 in each): Control group, TP group and TP-GL group. Pancreatic trauma was established with a custom-made biological impact machine-III, and GL was administered at 15 minutes after the accomplishment of operation. To determine survival rates during the first 7 days after injury, another 60 rats (n = 20 in each) were grouped and treated as mentioned above. At 24 hours of induction of TP, the histopathological changes in pancreas were evaluated and serum amylase levels were tested. Serum tumor necrosis factor α (TNF-α), interleukin 6 (IL-6), and HMGB1 were measured using enzyme linked immunosorbent assay. HMGB1 expressions in pancreas were measured using immunohistochemical staining, Western blot and Real-Time PCR analysis.

**Results:**

Serum levels of HMGB1, TNF-α and IL-6 were increased dramatically in TP group at 24 hours after induction of TP. However, these indicators were reduced significantly by GL administration in TP-GL group comparing with TP group (*P*<0.05). Meanwhile, survival analysis showed that the seven-day survival rate in TP-GL group was significantly higher than that in TP group (85% versus 65%, *P*<0.05). GL treatment significantly decreased the pancreatic protein and mRNA expressions of HMGB1 and ameliorated the pancreatic injury in rats with TP.

**Conclusions:**

Glycyrrhizin might play an important role in improving survival rates and ameliorating pancreatic injury of TP by suppression of the expressions of HMGB1 and other proinflammatory cytokine.

## Introduction

Although pancreatic trauma is rare, occurring in only 2% to 5% of trauma victims, it is often imperceptible and intractable with a higher morbidity and mortality. Most pancreatic injuries in China are due to blunt abdominal trauma, such as motor vehicle crashes, falls, bicycle handlebar injuries, etc., while in Western countries, pancreatic injuries are due to penetrating abdominal trauma. The incidence of pancreatic trauma accounts for 5% of closed abdominal trauma and 2%–6% of abdominal penetrating trauma [1]. As early signs and symptoms of pancreatic trauma are not obvious, it is often noticed until trauma-induced acute pancreatitis is presented. Trauma-induced acute pancreatitis, also referred to as traumatic pancreatitis (TP), are often followed by some serious complications, such as systemic inflammatory response syndrome (SIRS), shock, multiple organ failure (MOF), acute pancreatitis (AP), etc [2]. Although the pathogenesis and treatments of acute pancreatitis induced by other causes have been widely researched, there are few researches on the treatments of trauma-induced acute pancreatitis.

High mobility group box 1(HMGB1) is an intranuclear highly conserved nonhistone chromosomal protein that functions as a stabilizer of nucleosome structure and regulator of the genes transcription [3]–[4]. HMGB1 can be actively or passively released from cells and plays important roles in a large variety of diseases, such as trauma, burn, ischemia-reperfusion injury, sepsis, transplantation, surgical stress, shock, even in the cancer [5]–[8]. A strong correlation is found between HMGB1 levels and severity of AP, accordingly, it has been speculated that HMGB1 might be a target for anti-inflammatory treatment in AP [9]–[11]. Thus, inhibitors of HMGB1 were investigated to explore potential new treatment strategy for AP.

Recently, Glycyrrhizin (GL) was recognized as an HMGB1 inhibitor, which binds directly to HMGB1 and inhibits its cytokine activities [12]. GL, a main active ingredient in licorice root, is usually administered to treat patients with chronic hepatitis [13]. This compound is associated with numerous pharmacologic effects, including anti-inflammatory, antiviral, antitumor, and hepatoprotective activities [14]. However, the roles and mechanisms of GL in the treatment of AP, especially trauma-induced AP, were not investigated previously.

Accordingly, we hypothesize that glycyrrhizin may potentially improve the outcome of traumatic pancreatitis by inhibiting HMGB1. We have developed an experimental model of isolated traumatic pancreatitis [15] in rats and have been interested in the mechanisms and therapies of traumatic pancreatitis [16].

## Materials and Methods

### 2.1. Animals

Male Wistar rats (250 g±30 g) were purchased from the Experimental Animal Center, Third Military Medical University (Chongqing, China). All animals were bred and housed in standard cages in a climate controlled environment with an ambient temperature of 22±1°C and a 12-h light-dark cycle for 7 days before experiments. The animals were fed standard laboratory chow and water. The rats were fasted for 12 h before the experiment. Animals used in the present study were maintained in accordance with the guidelines of the Institutional Animal Care and Use Committee of the Third Military Medical University and ARRIVE (Animal Research: Reporting of In Vivo Experiments) guidelines. The protocol of the current study was approved by this committee (Permit Number: KY-2011065).

### 2.2. Establishment of Traumatic Pancreatitis Model

Traumatic pancreatitis was induced according to our previous reports with some modifications [15]. Briefly, all rats were anesthetized with an intraperitoneal injection of 2.5% sodium pentobarbital (30 mg/kg, Sigma, USA) before the operation. Then, the rat was fixed in the supine position onto a wooden board. The pancreas was completely exposed through a midline incision after shaving and disinfection. Next, a plastic shim was placed in the rear of the impact site. The pancreas was impacted using compressed air with a single 400 kPa pressure, which was produced by a custom-made biological impact machine-III (Daping Hospital, Third Military Medical University, Chongqing, China). After the impact, the pancreas was carefully put back and the abdomen was closed. All the rats were consecutively monitored every 6 hours and received meloxicam (Boehringer Ingelheim, France) via the tail vain (2 mg/kg once daily for 2 days) as a postoperative analgesic. The rat was allowed to drink water freely but could not eat anything for 24 hours after recovering from the anesthesia.

### 2.3. Experimental Design

A total of 60 rats were randomly divided into three groups (n = 20 in each): (1) sham operation control group (Control), in which animals underwent only laparotomy; (2) TP group; (3) TP-GL group, in which TP rats received GL. GL (0.2%, 6 mg/kg, saline as solvent, Minophagen Pharmaceutical Co, Tokyo, Japan) was administered intravenously via the tail vein at 15 minutes after the abdomen was closed. All the rats were killed humanely (2.5% sodium pentobarbital, 100 mg/kg, intraperitoneal injection) at 24 hour after injury to collect samples. To determine survival rates during the first 7 days after injury, another 60 rats (n = 20 in each) were grouped and treated as mentioned above. Euthanasia of rats is expected if rats demonstrate the one of the listed conditions: (1) Inappetance: complete anorexia for 24 hours; partial anorexia (less than 50% of caloric requirement) for 3 days. (2) Weakness/inability to obtain feed or water: inability or extreme reluctance to stand which persists for 24 hours, assuming that the animal has recovered from anesthesia. (3) Moribund state: measured by a lack of sustained purposeful response to gentle stimuli (example of purposeful response- weak attempt to get up; if rat is on its side, attempts should be asymmetrical in nature). (4) Infection: infection involving any organ system (either overt, or indicated by increased body temperature) and is accompanied by systemic signs of illness. (5) Signs of severe organ system dysfunction: such as severe vomiting or diarrhea, obstruction, peritonitis, anuria, oliguria, paralysis of one or more extremities, pain unresponsive to analgesic therapy, locomotor dysfunction, etc. Serum samples were separated from heart blood by centrifugation (1800 g for 15 minutes at 4°C) and stored in −20°C refrigerator. The pancreatic tissue was divided into two parts: one part was fixed in 10% neutral formalin for histopathological analysis and embedded in paraffin wax for cutting sections; another part was removed immediately, frozen and stored in liquid nitrogen.

### 2.4. Measurements of Serum Amylase Levels

The levels of serum amylase were determined using an enzyme-based colorimetric assay on a fully automated Hitachi 7170 biochemistry analyzer (Hitachi, Tokyo, Japan).

### 2.5. Determination of Tumor Necrosis Factor-α, Interleukin-6 Levels and HMGB1 in Plasma

The serum concentrations of tumor necrosis factor-α (TNF-α), interleukin-6 (IL-6) and HMGB1 were measured with commercially available enzyme-linked immunosorbent assay kits (TNF-α, IL-6: R&D Systems, Minneapolis, MN; HMGB1: Shino-Test Corp, Sagamihara, Japan) according to the instructions of the manufacturers.

### 2.6. Histopathological Analysis

The pancreas sections were stained with hematoxylin and eosin (H&E) and scored for edema, parenchymal necrosis, hemorrhage, and inflammation infiltration by two experienced pathologists who were blinded to the grouping and treatment according to Rongione's method [17] under a light microscope (CH20, Olympus, Japan). (a) Edema: 0 = null; 1 =  diffuse expansion of interlobular septa; 2 = 1 + diffuse expansion of interlobular septa; 3 = 2+interacinous space broadened; 4 = 3+ intercellular space broadened. (b) Necrosis: 0 =  null; 1 = 1 to 4 necrotic cells/high-power field; 2 = 5 to 10 necrotic cells/high-power field; 3 = 11 to 15 necrotic cells/high-power field; 4 = ≥16 necrotic cells/high-power field. (c) Hemorrhage: 0 =  null; 1 = 1 to 2 points; 2 = 3 to 5 points; 3 = 6 to 7 points; 4 = ≥8 points. (d) Inflammatory cell infiltration: 0 =  null; 1 =  around ductal margin; 2 =  in parenchyma <50% of lobules; 3 =  in parenchyma 51% to 75% of lobules; 4 =  in parenchyma >75% of lobules. Five fields of each section were counted, and the average scores of these 5 fields were the pathological injury scores of this section.

### 2.7. Immunohistochemical Analysis of HMGB1

Paraffin sections of pancreas tissue were de-waxed, rehydrated in gradient alcohol, and endogenous peroxidase activity was blocked with 3% H_2_O_2_ for 10 min. High-temperature antigen retrieval involved boiling the slides in citrate buffer (0.01 M, pH 6.0) for 20 minutes. The sections were incubated in normal goat serum at room temperature for 10 minutes and followed by incubation with polyclonal HMGB1 primary antibody (Biosynthesis biotechnology Co Ltd, Beijing) overnight at 4°C. At last, the sections were incubated with biotinylated secondary antibodies for 1 h at room temperature and then treated with streptavidin-peroxidase complex and visualized by incubating with diaminobenzidine (DAB) solution. Finally, sections were counterstained with hematoxylin.

### 2.8. Western Blot Analysis

Pancreatic protein was extracted by nuclear and cytoplasmic extraction reagents according to the manufacture's instructions (Beyotime Institute of Biotechnology, Shanghai, China). Protein concentration was determined using a commercial BCA protein assay kit (Pierce, Rockford, IL). For the immunoblotting analysis, proteins were separated by a 4% to 8% polyacrylamide gel and transferred by electrophoresis to polyvinylidene difluoride membranes (Millipore, Bedford, MA). For nonspecific bindings, membranes were blocked with 5% nonfat milk in TBS containing 0.1% Tween 20 overnight at 4°C. Then, the membranes were incubated with a diluted solution of anti-HMGB1 antibody (1∶200, Santa Cruz, CA) or anti-GAPDH antibody (1∶1000, Abcam, Cambridge, MA) at 4°C overnight. After incubation with the secondary antibody, anti-immunoglobulin G horseradish peroxidase conjugate (Bio-Rad, Hercules, CA), the membrane was exposed to a chemiluminescent reagent (Amersham Biotechnology Pharmacia, Piscataway, NJ). Specific protein bands were photographed, The band concentration was calculated by the quantification of the integrated optical density of the appropriate band using Quantity One software (Bio-Rad, Hercules, CA).

### 2.9. RNA extraction and Real-Time PCR

Total RNA was extracted with Trizol reagent (Invitrogen Corporation, Carlsbad, CA, USA) according to the manufacturer's instructions. The purity of RNA was verified by ethidium bromide staining on 1% agarose gels, and the integrity of RNA was verified by the presence of well-defined 28S and 18S rRNA bands. The purity of RNA was also quantified spectrophotometrically by a 260/280 ratio. Total cDNA was synthesized from the isolated total RNA using a reverse transcriptional system. Briefly, 5 µg of total RNA was reverse transcribed using 0.5 µg oligo (dT) 15 U avian myeloblastosis virus reverse transcriptase (Biouniquer Technology CO, LTD). The primers for quantitative real-time detection were as follows: 5′ -TGCTGCATATCGAGCTAAAGG- 3′ and 5′ - CCATACTGTACCAGGCAAGGT- 3′ for HMGB1 (399 bp), 5′ -ACGGTCAGGTCATCACTATCG- 3′ and 5′ - GGCATAGAGGTCTTTACGGATG- 3′ for β-actin (155 bp). The real-time PCR reaction was performed in LightCycler systems according to the manufacturer's instructions. In each PCR reaction of 2 µL complementary DNA, a final volume of 20 µL was used containing 0.8 µM of forward and reverse primers and 10 µL of SYBR Premix Ex Taq (TaKaRa). For relative quantification we used external standard curves. External standards were prepared by serial dilution (1∶10∧2 to 1∶10∧5) of cDNA. Melting curve analysis and electrophoresis on the agarose gel were used to ensure the specificity of the amplified products. The mRNA copy numbers of the transporter genes were normalized to those of rat β-actin.

### 2.10. Statistical Analysis

Statistical analyses were performed using SPSS Windows 17.0 statistical analysis software (Chicago, Ill). The data of serum cytokines are presented as median (interquartile range) and the other results are presented as mean ± standard deviation (SD). Groups were compared using Mann–Whitney U-tests. In the mortality study, time-to-survival data were analyzed by the Kaplan-Meier method and compared with the log-rank test. P values less than 0.05 was considered statistically significant.

## Results

### 3.1. Survival Analysis

**Figure 1:**
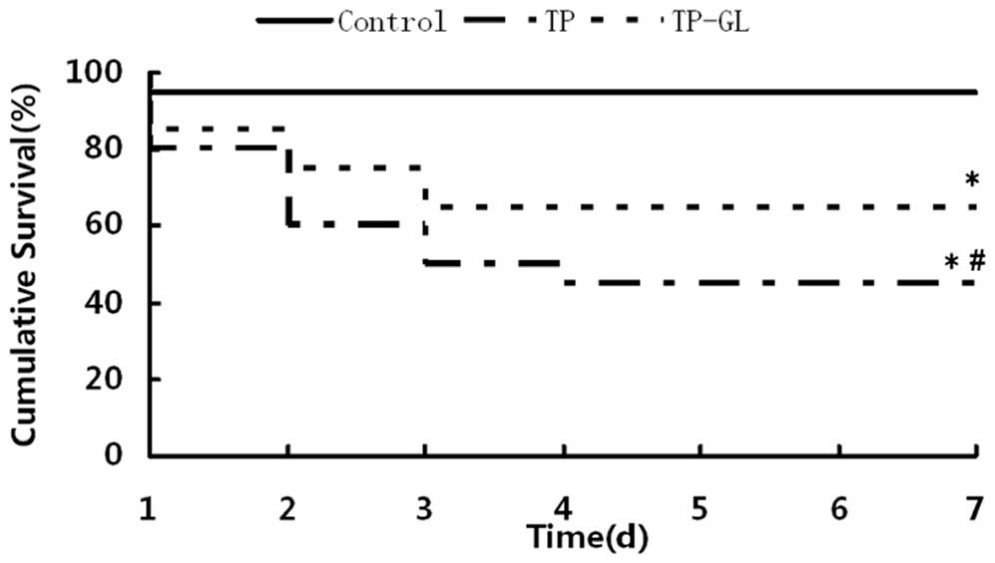
Survival analysis of TP rats. Kaplan-Meier survival curves of rats during 7 days after the induction of traumatic pancreatitis showed that the seven-day survival rate in the TP group was significantly lower than that in the TP-GL group (45% versus 65%), indicating that GL might participating in the improvement of the survival rate of TP rats. *P<0.05 versus Control group, # *P*<0.05 versus TP group. (TP, traumatic pancreatitis; GL, Glycyrrhizin).

The survival curves are shown in Fig. 1. Only one rat died in the first 24 h (probably due to anesthetic accident) and the rest survived in the next 6 days in the Control group. The survival rate over the first 24 h in the TP group was 80% (16/20 rats), and that in the TP-GL group was 85% (17/20 rats). The survival rate was 45% (9/20 rats) at 7 days after impact injury in the TP group, and that in the TP-GL group was 65% (13/20 rats). The final cause of early death for the rats was abdominal bleeding and the late was abdominal infections. Seven-day survival of the TP-GL group was significantly higher than that of the TP group (*P*<0.05, Fig. 1), indicating that GL might be involved in the improvement of the survival rate of TP rats.

### 3.2. Serum Amylase Levels

**Figure 2:**
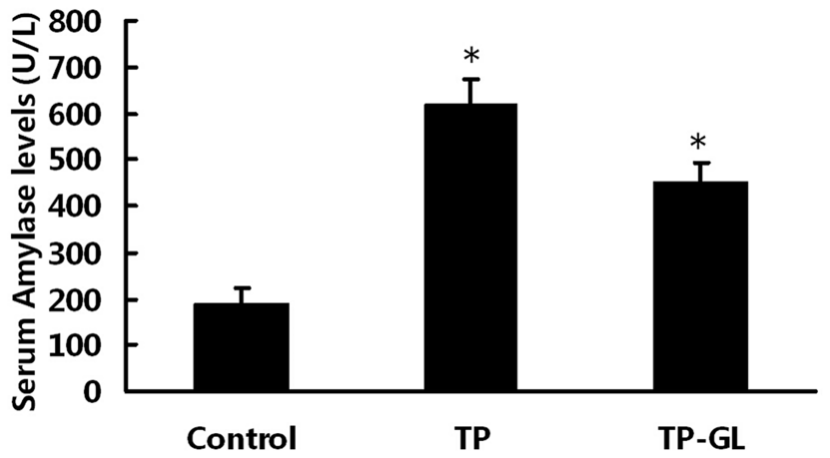
Serum levels of amylase in rats. At 24 h after induction of impact injury, serum amylase levels of TP group and TP-GL group were higher than those of the Control group. The serum amylase levels in TP-GL group were slightly lower than those in TP group, but there were no statistical significance.* *P*<0.05 versus Control group. (TP, traumatic pancreatitis; GL, Glycyrrhizin).

At 24 h after induction of impact injury, serum amylase levels of TP group and TP-GL group were higher than those of the Control group. The increased amylase levels of two groups confirmed the effectiveness of this compressed air impact induced TP. The amylase levels of TP-GL group were slightly lower than those in TP group, but there were no statistical significance. This finding indicated that GL had no inhibitory effect on pancreatic enzymes (Fig. 2).

### 3.3. Serum Tumor Necrosis Factor-α and Interleukin-6 Levels

**Figure 3:**
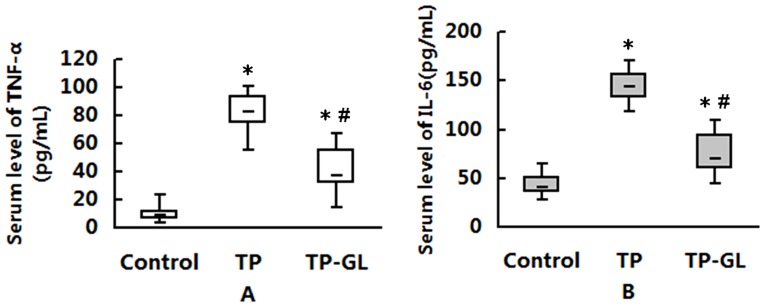
Serum levels of TNF-α and IL-6 in rats. At 24 hours after impact injury, serum levels of TNF-α (A) and IL-6 (B) in TP and TP-GL group were remarkably higher than those in Control group. Meanwhile, both of the two proinflammatory cytokines revealed a significant decrease in TP-GL group compared with TP group. * *P*<0.05 versus Control group, # *P*<0.05 versus TP group. (TP, traumatic pancreatitis; GL, Glycyrrhizin).

Serum TNF-α and IL-6 levels were measured at 24 hours after impact injury. Remarkably high levels of TNF-α and IL-6 were found in TP and TP-GL group, compared with Control group (*P*<0.05). Meanwhile, both of the two proinflammatory cytokines revealed a significant decrease in TP-GL group compared with TP group (*P*<0.05, Fig. 3). These results suggested that GL might play a potential role in inhibiting the inflammatory reaction during trauma-induced acute pancreatitis.

### 3.4. Serum HMGB1 Levels

**Figure 4:**
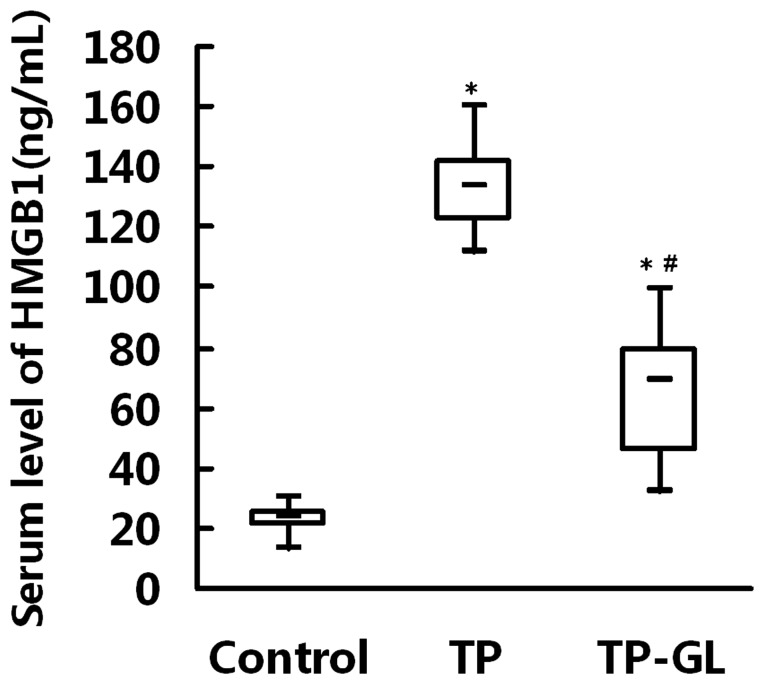
Serum levels of HMGB1 in rats. At 24 h after induction of impact injury, the serum levels of HMGB1 in TP and TP-GL groups were significantly higher than those in Control group. Moreover, the serum HMGB1 levels of TP-GL group showed obvious reduction compared with TP group after 24 h of impact injury. * *P*<0.05 versus Control group, ^#^ *P*<0.05 versus TP group. (TP, traumatic pancreatitis; GL, Glycyrrhizin).

The marked elevation of serum HMGB1 levels was observed in TP and TP-GL group compared with Control group (*P*<0.05). Moreover, the serum HMGB1 levels of TP-GL group showed obvious reduction compared with TP group (*P*<0.05, Fig. 4). These findings indicated that GL administration might be associated with the inhibition of HMGB1 release during trauma-induced acute pancreatitis.

### 3.5. Pancreatic Histological Scores of Injured Pancreas

**Figure 5:**
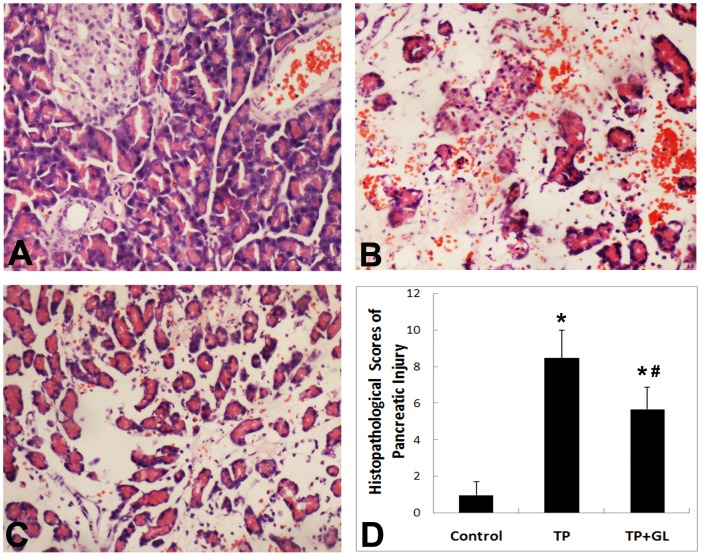
Effects of GL on pathological changes in pancreatic tissues. A: Pancreatic H&E (×200) stained sections. The normal architecture was found in sections from the Control group. In TP and TP-GL group, edema, necrosis, hemorrhage and inflammation were found obviously in the pancreatic tissue after 24 h of impact injury. However, these pathological damages in TP-GL group (C, H&E ×200) were significantly lighter than those in TP group (B, H&E ×200). Histopathologic scores were significantly higher in TP and TP-GL group comparing to Control group; however, the score of TP-GL group was less than that of TP group (D). * *P*<0.05 versus Control group, # *P*<0.05 versus TP group. (TP, traumatic pancreatitis; GL, Glycyrrhizin).

In TP and TP-GL group, edema, necrosis, hemorrhage and inflammation were found obviously in the pancreatic tissue (Fig. 5B and 5C). However, these pathological damages showed lighter in TP-GL group than those in TP group. Histopathologic scores were significantly higher in TP and TP-GL group; however, the scores of TP-GL group were less than TP group. Histopathological examination of the pancreas revealed that GL ameliorated TP injury in TP-GL group (Fig. 5D).

### 3.6. Effect of GL on HMGB1 Expression in Pancreas

**Figure 6:**
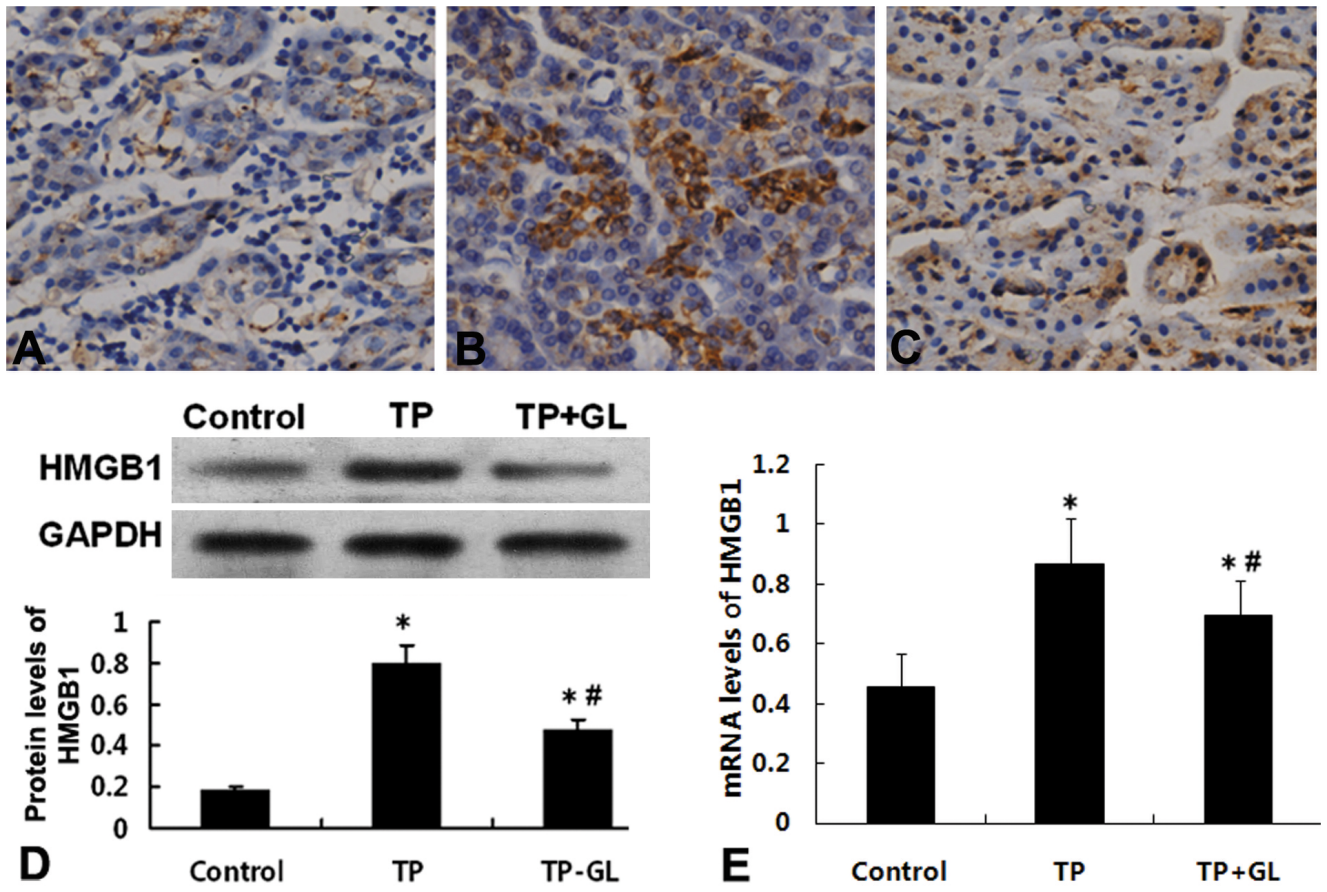
Effects of GL on the expressions of HMGB1 in rat pancreatic tissue. Pancreatic Immunohistochemical analysis showed that HMGB1 was slightly expressed in pancreatic tissue of Control group (A, IHC×200). After 24 h of impact injury, the expressions of HMGB1 were significantly increased (B, IHC×200). Meanwhile, the administration of GL significantly reduced the expression of HMGBI in TP rats (C, IHC×200). Western blot also revealed the same pattern of changes of HMGB1expression in pancreatic tissue of the three groups (D). Real-Time PCR also showed that the mRNA levels in TP rat were significantly higher than those in rats of Control group, and were reduced by the administration of GL (E). * *P*<0.05 versus Control group, # *P*<0.05 versus TP group. (TP, traumatic pancreatitis; GL, Glycyrrhizin).

Immunohistochemical analysis showed that HMGB1 was slightly expressed in pancreatic tissue in Control group (Fig. 6A), strongly expressed in the TP group (Fig. 6B), and expressed at an intermediate level in the GL-treated group (Fig. 6C). Meanwhile, Western blot analysis revealed that the expression of TP and TP-GL group is higher than the Control group; however, compared to TP group, HMGB1 expression levels in the TP-GL rats were evidently lower than those at 24 h after impact injury (Fig. 6D). Likewise, Real-Time PCR analysis showed that among the GL treated rats, HMGB1 expression levels were evidently lower than those in the TP rats at 24 h after impact injury (Fig. 6E). These results indicated that GL administration might be associated with the suppression of HMGB1 expression in pancreatic tissues during trauma-induced acute pancreatitis.

## Discussion

As pancreatic injury is a rare complication during abdominal trauma, there are few researches focused on this disease. However, its higher morbidity and mortality prompt us to further investigate the pathogenesis and treatment strategy to improve its outcome. First of all, it is necessary to establish an animal model of TP for elucidating the pathogenesis and exploring potential effective treatment strategy for TP. For simulating clinic situation, our team developed a controllable rat model of TP and used a controlled compressed air to impact pancreas at a certain pressure [15]. Our new TP rat model was considered to closely simulate the pathogenesis of isolated TP without injury to other adjacent organs and can analyse the pancreas injury in different quantitative impact pressures. As a result, we believe that this model is superior to Modlin's non-crushing vascular clamp method and Delany's falling weight technique [18]–[19].

Through decades of investigation, AP has been considered as a life-threatening inflammatory disease, as the inflammatory mediator theory has indicated that the abnormal activation of pancreatin triggers the inflammatory cells and they release proinflammatory cytokines in the early stage of AP [20]. However, the relationship between pancreatic trauma and the consequent uncontrolled systemic inflammatory response has remained elusive. Increasing evidence from recent studies has implicated that the inflammatory mediators and proinflammatory cytokines also play pivotal roles in the pathogenesis of TP and subsequent local and systemic complications. These inflammatory cytokines, leading to SIRS, MOF and even death, are associated with the severity of severe acute pancreatitis (SAP). Among them, TNF-α and IL-6 plays key roles in the pathogenesis of SAP and trauma [7], [21]–[22]. However, some studies found that levels of TNF-α, IL-1β, IL-6 in SAP or sepsis reached to a peak in the early several hours and then underwent subsequent decrease towards normal levels, while the inflammatory response and organs injury still sustained, indicating that some late proinflammatory mediators might contribute to the pathogenesis of SAP and sepsis. Consequently, the therapies of anti-TNF-α, IL-1β, and IL-6 were proved to be limited and disappointing [23]–[24], while it might be a promising strategy to explore new treatments targeted on the late proinflammatory mediators.

Unlike other proinflammatory cytokines, HMGB1 was recognized as a late-appearing inflammatory mediator, and it is secreted at peak about 20 hours after stimulation [25]–[27]. HMGB1 can bind to the receptor for advanced glycosylation end product (RAGE), Toll-like receptor 2 (TLR2), and Toll-like receptor 4 (TLR4) to enhance the inflammatory response [28]–[30]. HMGB1 was found to be up-regulated in many acute and chronic diseases [6]–[8] including SAP. Yasuda measured serum HMGB1 concentrations in 45 patients with SAP at the time of admission and found that the mean value of serum HMGB1 levels was significantly higher in patients with SAP than that in healthy volunteers. Also, Serum HMGB1 levels were significantly positively correlated with the Japanese severity score and Glasgow score. These results suggest that HMGB1 may act as a key mediator for inflammation and organ failure in SAP [9]. Cheng and his colleagues measured serum HMGB1 levels in rat models of SAP and found that serum HMGB1 levels were not significantly altered for the first 12 hours after SAP was induced. However, HMGB1 increased dramatically after 12 hours and reached the peak at 24 hours, on the basis of which our present study chose 24 h after impact as the detection time. Meanwhile, it was observed that HMGB1 could remain at a relatively high level for 72 hours [11]. As a result, compared to other proinflammatory cytokines, this characteristic of HMGB1 with delayed presence provides a wide and effective therapeutic window and become a unique target for anti-inflammatory therapy [31]–[32]. Therefore, inhibition of HMGB1 secretion or release becomes a new therapy method of TP.

Glycyrrhizin (GL), a natural compound of triterpene glycoside, is extracted from the licorice root which is widely cultivated throughout Europe and Asia and has been used medically for at least 2, 500 years. Glycyrrhizin is commonly used in treating patients with liver diseases based on its anti-inflammatory and antiviral effects [33]. More recently, some studies indicated that GL could directly bind to HMGB1 protein by interaction with two arms of both HMG boxes and inhibited its cytokine activities by inhibition of HMGB1 chemoattractant and mitogenic activities [12]. Moreover, GL could reduce the serum level and gene expression of HMGB1 and other proinflammatory cytokines and protect vital organs against porcine endotoxemia [24]. Our present study indicated that the glycyrrhizin was beneficial for the management of TP. As far as we know, the current study is the first report on the effect of GL in the treatment of TP.

In the present study, we found that GL can not only reduce the serum levels of TNF-α and IL-6, which were previously reported to reach to a peak in the early several hours, but also decrease the serum level of HMGB1 in rats at 24 hours after induction of TP. Moreover, it was showed that GL could also significantly inhibit the expression of HMGB1 in pancreas of TP. Although it has been reported that GL could suppress the proinflammatory activities of HMBG1, the mechanisms by which GL inhibited the expression of HMBG1 in local tissues or peripheral blood remained to be unclear. We presumed that the inhibition of HMGB1 expression might be associated with the alleviation of tissue inflammatory injuries after GL administration, as GL could extenuate the inflammatory reaction by inhibiting the activities of HMGB1 and other proinflammatory mediators. According to our present study, GL treatment obviously ameliorated pancreatic tissue injury and reduced the lethality of TP in rats. This finding suggested that GL might also exert its therapeutic effects on TP as HMGB1 inhibitor to extenuate the inflammatory reaction. However, the exact molecular mechanisms by which GL inhibits the expression of HMGB1 should be further elucidated.

In conclusion, the findings from our study indicate that glycyrrhizin can suppress HMGB1 and improve outcomes of traumatic pancreatitis in rats. Nevertheless, the definite mechanisms are still poorly understood. To clarify this, further basic and clinic investigations are required in the future.
